# Identification of Bacterial Membrane Selectivity of Romo1-Derived Antimicrobial Peptide AMPR-22 via Molecular Dynamics

**DOI:** 10.3390/ijms23137404

**Published:** 2022-07-03

**Authors:** Hana Kim, Young Do Yoo, Gi Young Lee

**Affiliations:** 1 Laboratory of Molecular Cell Biology, Graduate School of Medicine, Korea University College of Medicine, Korea University, Seoul 02841, Korea; hnkim8381@korea.ac.kr; 2 Department of Microbiology and Immunology, Cornell University, Ithaca, NY 14853, USA

**Keywords:** AMPR-22, antimicrobial peptide, molecular dynamics, extensively drug-resistant bacteria, bacterial membrane selectivity

## Abstract

The abuse or misuse of antibiotics has caused the emergence of extensively drug-resistant (XDR) bacteria, rendering most antibiotics ineffective and increasing the mortality rate of patients with bacteremia or sepsis. Antimicrobial peptides (AMPs) are proposed to overcome this problem; however, many AMPs have attenuated antimicrobial activities with hemolytic toxicity in blood. Recently, AMPR-11 and its optimized derivative, AMPR-22, were reported to be potential candidates for the treatment of sepsis with a broad spectrum of antimicrobial activity and low hemolytic toxicity. Here, we performed molecular dynamics (MD) simulations to clarify the mechanism of lower hemolytic toxicity and higher efficacy of AMPR-22 at an atomic level. We found four polar residues in AMPR-11 bound to a model mimicking the bacterial inner/outer membranes preferentially over eukaryotic plasma membrane. AMPR-22 whose polar residues were replaced by lysine showed a 2-fold enhanced binding affinity to the bacterial membrane by interacting with bacterial specific lipids (lipid A or cardiolipin) via hydrogen bonds. The MD simulations were confirmed experimentally in models that partially mimic bacteremia conditions in vitro and ex vivo. The present study demonstrates why AMPR-22 showed low hemolytic toxicity and this approach using an MD simulation would be helpful in the development of AMPs.

## 1. Introduction

We are living in a new era where infectious diseases can negatively impact human life. An under-estimation of infectious pathogens can cause uncontrollable situations, alerting us to be prepared for the next pandemic. One of the possible pandemic scenarios is emergence of extensively drug-resistant (XDR) ESKAPE pathogens (*Enterococcus faecium*, *Staphylococcus aureus*, *Klebsiella pneumoniae*, *Acinetobacter baumannii*, *Pseudomonas aeruginosa*, and *Enterobacter* species) triggered by the abuse of antibiotics [1,2]. Only last-resort antibiotics, such as Colistin, are available for treating XDR. However, its narrow therapeutic area against Gram-negative pathogens and the severe off-target effects such as nephrotoxicity have restricted its use in life-threatening diseases such as XDR Gram-negative bacterial sepsis [3]. There is, therefore, an urgent need for antibiotics with a novel mechanism to overcome XDR [4]; however, antibiotic development has slowed, exacerbating the current situation.

To overcome the limitation of chemical-based antibiotics prone to attenuation by bacterial antibiotic resistance genes, antimicrobial peptides (AMPs) have been considered as novel antibiotics to treat XDR. They are host defense peptides derived from diverse living organisms, consisting of 5 to 100 amino acids [5]. By May 2022, over 22,400 natural and synthetic AMPs have been developed, over 16,100 AMPs have been patented, and 77 of them are under drug development according to the Data Repository of Antimicrobial Peptides (DRAMP) database (http://dramp.cpu-bioinfor.org (3 May 2022)) [6]. The classification of AMPs can be based on (1) structural characteristics (e.g., cationic amphipathic peptides or macrocyclic peptides) or (2) mechanisms of action (e.g., direct antimicrobial activity such as membrane disruption through pore formation, inhibition of protein synthesis, or immunomodulation by releasing pro-inflammatory cytokines) [7]. AMPs generally have a wide spectrum of antimicrobial activity against both Gram-positive and Gram-negative bacteria including XDR strains in conjunction with a high fitness cost required for resistance acquisition contrasting to chemical-based antibiotics, making them an appropriate solution for XDR [8,9].

AMPs with the potential to form pores were proposed to be one of the strategies for the novel development of antibiotics [10]. Reactive oxygen species modulator 1 (Romo1) is known to play a crucial role in cancer cell invasion [11], inflammation [12], cellular senescence [13], mitochondrial protein translocation [14], mitochondrial dynamics [15], and mitochondrial redox sensing [16]. Recently, Romo1 was proposed to be a viroporin-like non-selective channel that depolarizes mitochondria, leading to the design of AMPR-11 with the amphipathic pore-forming region of Romo1 [17]. AMPR-11 showed broad-spectrum antimicrobial activity against both Gram-negative and Gram-positive bacteria including clinically isolated multi-drug resistant (MDR) strains [18]. In contrast to magainin 2, with an efficacy that is restricted to Gram-negative bacteria, or cyclic lipopeptide daptomycin, restricted to Gram-positive bacteria, a single dose (10 mg/kg) of AMPR-11 administered intravenously showed a broad antimicrobial spectrum in a sepsis murine model triggered by either MDR Gram-positive or Gram-negative bacteria. Moreover, AMPR-11 did not show any in vivo toxicity even at a ten-fold effective concentration (100 mg/kg). To improve the efficacy of AMPR-11, AMPR-22 was constructed by replacing the polar residues (2THR, 5GLN, 6SER, and 9THR) in an amphipathic helix with lysine [19] and deleting 18MET. Compared to AMPR-11, AMPR-22 showed an enhanced antimicrobial effect in a murine model of sepsis induced by MDR bacteria with decreased hemolysis [20], which is a well-known limitation of intravenously administered AMP for therapeutics. How AMPR-22 shows higher efficacy and lower hemolytic toxicity over AMPR-11 has not been established, and this limits the optimization strategy.

One of the strategies for peptide optimization is a molecular dynamics (MD) simulation, a real-time computation of the movements of atoms in a limited three-dimensional space for dissecting the atomic mechanisms [21]. The computational power required for MD simulations increases exponentially with increase in three-dimensional space (a.k.a. periodic boundary condition), where the parallel calculation occurs. Therefore, since 2000, there has been a trend in using MD for studying AMP (56 cases by 2019) as the simulation requires a small periodic boundary condition [22]. Lipid dynamics is a well-studied area that explains how AMPs interact with lipids, which can affect antimicrobial activity. For example, magainin-H2, an AMP extracted from the skin of *Xenopus laevis*, bound on the surface of the lipid bilayer, forms disordered toroidal pores in the DPPC (1,2-dipalmitoyl-sn-glycero-3-phosphocholine) membrane [23]. LL-37, which is derived from the human cathelicidin family, was shown to preferentially bind POPG (1-palmitoyl-2-oleoyl-sn-glycero-3-phosphoglycerol), mimicking bacterial membranes, over POPC (1-palmitoyl-2-oleoyl-sn-glycero-3-phosphocholine), mimicking mammalian membranes [24]. MD simulations of two battacin analogs, whose toxicity is attenuated compared to the parent, showed how the positively charged amino acids and hydrophobic moieties play a role in the interaction with the inner membrane of *S. aureus* or that of *E. coli* [25]. MD simulation has become a fascinating tool to understand how AMPs act at the atomic level with a multi-scale view of a fundamental biophysical process (e.g., the complex interplay between AMPs and membrane, and what amino acids are involved in the membrane affinity) [26] as the computational power increases. 

Here, we used MD simulations to explore how AMPR-22 acts on membranes with low hemolytic toxicity. The results were confirmed by artificial membranes, mimicking bacterial inner/outer membranes or eukaryotic plasma membrane. We propose that MD simulation-guided AMP optimization could be a cost-effective strategy for development of a peptide drug.

## 2. Results

### 2.1. Construction of the Representative AMPR-11 or AMPR-22 Structure via Molecular Dynamics

**Figure 1:**
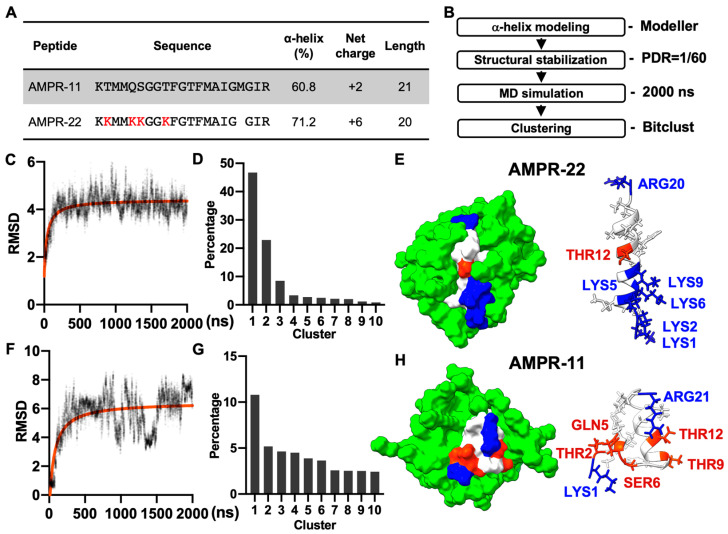
MD simulation of AMPR-11/22 in DPC micelles. (**A**) The amino acid sequence and chemicophysical properties of AMPR-11/22. The alpha helix proportion was calculated using the values from circular dichroism (CD) via the BeStSel server. (**B**) Experimental workflow for structure determination through MD in DPC micelles [Peptide detergent ratio (PDR) is 1/60]. (**C**,**F**) RMSD fluctuations of AMPR-22/11 by time. (**D**,**G**) Clustering of AMPR-22/11 structures in 0.5–2 µs (15,000 frames). (**E**,**H**) Representative structure of AMPR-22 or AMPR-11 reconstituted with DPC micelles. The hydrophobic (nonpolar), hydrophilic (polar), and cationic residues are shown in white, red, and blue, respectively. RMSD, root mean square deviation.

AMPR-11 is composed of 21 amino acids (K58-R78 region of Romo1) and AMPR-22 was constructed by substituting four polar amino acids (2THR, 5GLN, 6SER, and 9THR) with lysine and deleting 18MET to increase both amphipathicity and net positive charge (Figure 1A). Both AMPR-11 and AMPR-22 predominantly formed alpha-helical structures in circular dichroism (CD) (60.8% for AMPR-11 and 71.2% for AMPR-22) [18,20] when they were both dissolved in 50% hexafluoro-2-propanol (HFIP), which has been used for a membrane mimetic environment [27]. 

As AMPR-11 and AMPR-22 are intermediate AMPs that need to be optimized further, we performed a cost-effective MD simulation to predict their representative structures instead of using nuclear magnetic resonance (NMR) spectroscopy. Based on the alpha-helical proportion determined by the CD [18,20], the initial structures of AMPR-11 and AMPR-22 were modeled using Modeller. Each structure was simulated for 2 µs under a CHARMM36m force field with 60 molecules of dodecylphosphocholine (DPC) micelles, which have been widely used to solubilize amphipathic peptides or hydrophobic proteins for NMR (Figure 1B). The AMPR-22 (Figure 1C,D) or AMPR-11 (Figure 1F,G) structures in 0.5–2 µs were used for clustering to find the representative structure. The representative structure of AMPR-22 (Figure 1E) or AMPR-11 (Figure 1H) in the top cluster showed 79.0% or 50.0% alpha-helical proportion, respectively, comparable with the values acquired from the CD.

### 2.2. AMPR-11 and AMPR-22 Interact with Bacterial Inner/Outer Membranes Specifically over Eukaryotic Plasma Membranes

**Figure 2:**
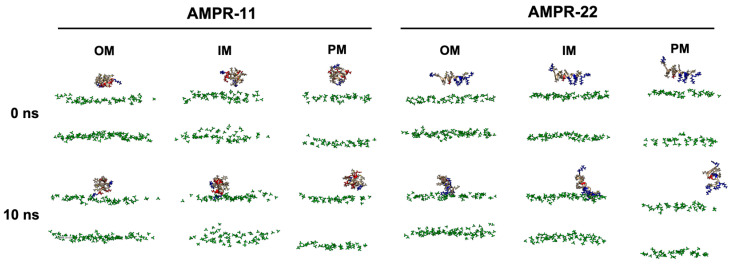
AMPR-11/22 interactions with Gram-negative bacterial outer membrane (OM), inner membrane (IM), or eukaryotic plasma membrane (PM). Each iteration having the average value of the hydrogen bonds formed between AMPR-11/22 and the membrane was selected: 25th (OM), 43rd (IM), and 43rd (PM) simulations from 100 iterations were selected for AMPR-11, and 1st (OM), 42nd (IM), and 48th (PM) simulations from 100 iterations were selected for AMPR-22. The phosphate group of the lipids is colored green. Hydrophobic (nonpolar), hydrophilic (polar), and cationic residues of AMPR-11/22 are shown in copper, red, and blue, respectively.

To determine whether there is a difference in the binding pattern of AMPR-11 or AMPR-22, their representative structures were used to simulate interactions with bacterial or eukaryotic membranes. Although either AMPR-11 or AMPR-22 was effective in a murine model of sepsis induced by MDR strains, AMPR-22 was more potent with lower hemolytic toxicity than AMPR-11. To clarify how this works at an atomic level, we constructed membranes mimicking the Gram-negative bacterial outer membrane (OM), inner membrane (IM), or the eukaryotic plasma membrane (PM) for an all-atom (AA) MD simulation (Table 1). One AMPR-11 or AMPR-22 was placed onto the membrane to observe the peptide interactions with each membrane. We first performed 10 ns simulations with 100 iterations to identify the initial contact of AMPR-11/22 with each model membrane. Both AMPR-11 and AMPR-22 (Figure S1) bound to the bacterial OM and IM in 4 ns with high affinity, and the peptides were not disassociated until the last 10 ns. On the contrary, neither AMPR-11 nor AMPR-22 could bind tightly to the eukaryotic PM (Figure 2).

**Figure 3:**
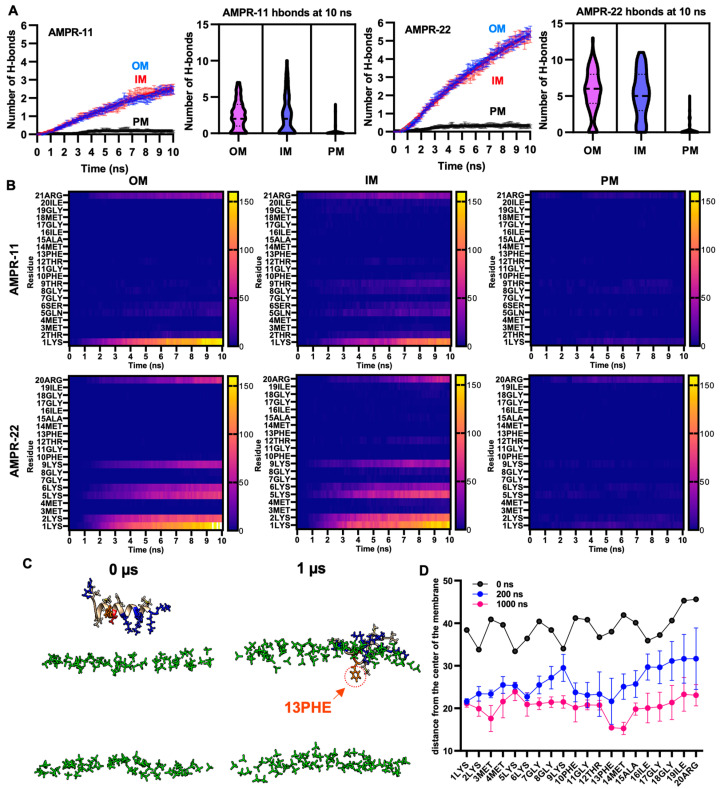
Hydrogen bond formation between AMPR-11 or AMPR22 and model membranes. (**A**) The number of hydrogen bonds of AMPR-11 or AMPR-22 with each model membrane was calculated from 100 iterations. (**B**) Heatmap describing hydrogen bonds formed between each amino acid of AMPR-11 or AMPR-22 and the indicated model membrane. (**C**,**D**) MD simulation of AMPR-22 for 1 μs, (**C**) 42nd simulation (IM) from 100 iterations was used to examine its insertion into the model membrane. The phosphate group of the lipids is colored green. Hydrophobic (nonpolar), hydrophilic (polar), and cationic residues of AMPR-11/22 are shown in copper, red, and blue, respectively; 13PHE is colored in orange and marked with an arrow. (**D**) Distance of each amino acid of AMPR-22 from the middle plane of the model membrane was plotted at 0, 200, and 1000 ns. Data represent average ± standard error of the mean.

To quantify interaction patterns, we analyzed the number of hydrogen bonds—one of the strongest intermolecular interactions—formed between AMPR-11 or AMPR-22 and each model membrane. There was a significant increase in the number of hydrogen bonds in the bacterial membrane compared to the plasma membrane (Figure 3A). Moreover, the total number of hydrogen bonds between AMPR-22 and the bacterial membrane was two-fold higher than that of AMPR-11, and either AMPR-11 or AMPR-22 formed 17.7- or 21.3-fold more hydrogen bonds with bacterial membrane than with eukaryotic membrane, respectively. This indicates that AMPR-22 has a higher bacterial membrane preference over AMPR-11 and might explain the previously reported higher efficacy of AMPR-22 compared to AMPR-11 [20].

To identify essential amino acids corresponding to increased bacterial membrane preference, we analyzed hydrogen bonds formed between the amino acids of AMPR-11 or AMPR-22 and the lipids of the bacterial membrane or eukaryotic plasma membrane [28,29]. The consensus of dominant interactions was in positions 1, 2, 5, 6, 9, and the last residues (21st of AMPR-11, 20th of AMPR-22). In AMPR-11, strong binding affinity was shown in 1LYS and 21ARG, and a weak/moderate affinity was shown in 2THR, 5GLN, 6SER, and 9THR, which are located on the polar surface in the amphipathic helix. Lysine residues (2LYS, 5LYS, 6LYS, and 9LYS) of AMPR 22 formed 13.7- or 5.1-fold more hydrogen bonds with bacterial OM or IM compared to the corresponding polar residues (2THR, 5GLN, 6SER, and 9THR) of AMPR-11, respectively (Figure 3B). This could be associated with the better antimicrobial activity of AMPR-22 over AMPR-11.

We selected one of the iterations for AMPR-22 (42nd simulation of 100 iterations for inner membrane) that could represent an average number of hydrogen bonds and simulated it for 1 µs. Membrane stability was monitored via tracking area per lipid and membrane thickness by time (Figure S2). Although we were not able to see the penetration of AMPR-22 into the bacterial inner/outer membranes in three iterations, 13PHE and 14MET were inserted deeply into the membrane compared to other residues over time (Figure 3C,D). This result is consistent with the report showing that antimicrobial activity was significantly decreased or lost if 13PHE was deleted from AMPR-11 [20]. Additionally, we performed a coarse-grained (CG) simulation for 1 ms by placing nine AMPR-22 onto the bacterial inner membrane parallelly under a martini22p force field to determine penetration and/or pore formation events. However, neither penetration of AMPR-22 nor pore formation was detected in three iterations (Figure S3). The results may imply that penetration could occur at a later stage than believed (e.g., multi-ms to multi-s).

### 2.3. AMPR-22 Permeabilizes Bacterial Membrane Mimicking Large Unilamellar Vesicles (LUVs) by Selectively Binding to Cardiolipin or Lipid A

We examined which lipid component of the bacterial inner/outer membranes corresponds to initial contacts with AMPR-11/22. We found that AMPR-22 formed more hydrogen bonds with cardiolipin in the inner membrane even though there is a significantly smaller proportion of cardiolipin compared to phosphatidylethanolamine (PE) and phosphatidylglycerol (PG) (Table 2). The preference of AMPR-11/22 to lipid A was not evaluated as the outer leaflet of the outer membrane was exclusively composed of lipid A.

**Figure 4:**
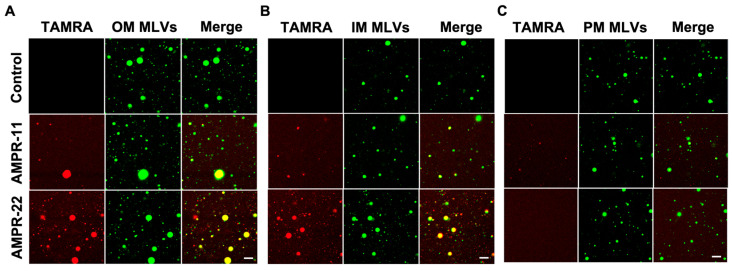
Interaction of AMPR-11/22 with the model membranes. Artificial MLVs containing 0.5% NBD-PE (molar ratio) were incubated with 5 μg/mL TAMRA-labeled AMPR-11/22 for 10 min. AMPR-11/22 interaction with the bacterial OM (**A**), IM (**B**), or PM (**C**) was monitored via confocal microscopy. Scale bar, 20 μm.

We next examined whether AMPR-11/22 bound selectively to the bacterial membrane through the following in vitro experiments. To mimic biological membranes, we generated large multilamellar vesicles (MLVs) suspension with dehydration/rehydration technique. NBD-PE [1,2-dioleoyl-sn-glycero-3-phosphoethanolamine-N-(7-nitro-2-1,3-benzoxadiazol-4-yl)] was added to visualize MLVs. Bacterial IM, OM, or eukaryotic PM-mimic MLVs was generated with the same composition as used in MD simulations (Table 1). Carboxytetramethylrhodamine (TAMRA)-labeled AMPR-11 or AMPR-22 was synthesized to visualize its adhesion and was incubated with each model MLVs. AMPR-22 showed a higher binding affinity to the bacterial OM-MLVs (Figure 4A) or IM-MLVs (Figure 4B) than did AMPR-11. In contrast, AMPR-22 did not bind at all to the PM-MLVs, and AMPR-11 appeared to bind weakly (Figure 4C). These results indicate that AMPR-22 selectively binds to the bacterial membrane without interacting with the eukaryotic plasma membrane.

**Figure 5:**
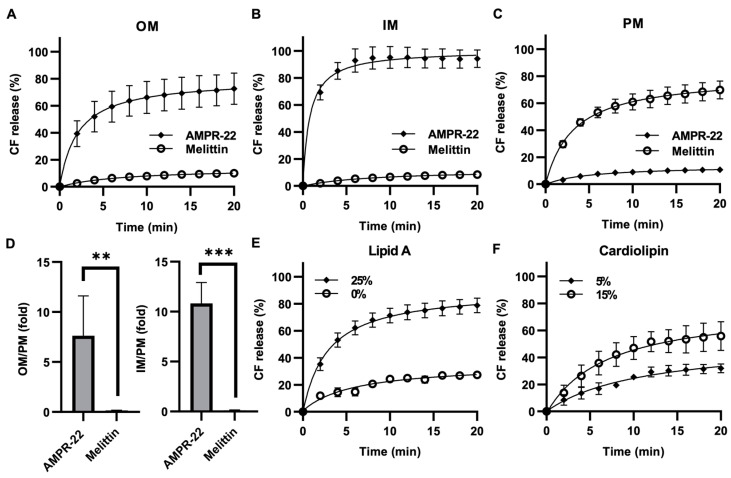
AMPR-22-induced membrane permeabilization. CF-encapsulated OM-LUVs (**A**), IM-LUVs (**B**), or PM-LUVs (**C**) were constructed using the lipid composition used for MD. The LUVs were incubated with 0.1% HFIP (negative control), 1% Triton X-100 (positive control), 2 μM AMPR-22, or 0.05 μM Melittin. (**D**) The membrane permeabilization of OM-LUVs (left panel) or IM-LUVs (right panel) was divided by that of PM-LUVs, representing the membrane preference of AMPR-22. Membrane permeabilization of OM-LUVs with additional lipid A (**E**) or IM-LUVs with additional cardiolipin (**F**). Bars represent average ± standard deviation of three independent experiments. ** *p* < 0.01, *** *p* < 0.001 by unpaired t tests.

Because AMP permeabilizes the membrane through pore formation, to evaluate AMPR-22-induced liposome permeabilization, we generated carboxyfluorescein (CF)-encapsulated LUVs composed of the lipid composition listed in Table 1. AMPR-22 at 2 µM induced CF leakage: 73% in OM-LUVs (Figure 5A), 94% in IM-LUVs (Figure 5B), and 11% in PM-LUVs (Figure 5C). On the contrary, a pore-forming toxin, melittin, induced 70% CF release in PM-LUVs, even at 0.05 µM (Figure 5C). The AMPR-22-induced CF leakage in OM versus PM or IM versus PM was calculated to be 7.6- or 10.8-fold, respectively (Figure 5D). Moreover, AMPR-22-induced CF leakage was lipid A (Figure 5E)- or cardiolipin (Figure 5F)-dependent, similar to its lipid preference shown in MD simulations (Table 2). These results indicate that AMPR-22 targets cardiolipin of the IM or lipid A of the OM to induce bacterial membrane permeabilization. The high bacterial membrane affinity can reduce the potential of off-target effects, such as hemolytic toxicity, which is one of the critical factors in evaluating intravenously administered drug candidates. 

### 2.4. AMPR-22 Selectively Binds to Carbapenem-Resistant P. aeruginosa (CRPA) in Bacteremia-Mimic Conditions

As AMPR-22 was designed to administer intravenously for treating MDR/XDR-triggered sepsis, the hemolytic toxicity should be considered in conjunction with its efficacy, and AMPs for intravenous injection should target bacteria specifically over red blood cells (RBCs). We thus evaluated whether AMPR-22 selectively binds to bacteria in a mixture of mice RBCs and clinically isolated MDR CRPA [30], whose condition partially mimics bacteremia of infected individuals.

**Figure 6:**
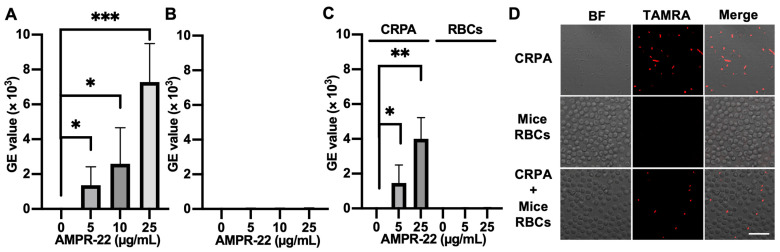
Interaction of TAMRA-labeled AMPR-22 with carbapenem-resistant *P. aeruginosa* (CRPA) or mouse red blood cells (RBCs). CRPA, mouse RBCs, or CRPA/RBCs group was incubated with TAMRA-labeled AMPR-22 for 10 min and analyzed via flow cy-tometry (**A**–**C**) or confocal microscopy (**D**). Bars represent average ± standard deviation of three independent experiments. * *p* < 0.05, ** *p* < 0.01, *** *p* < 0.001 by unpaired t tests. BF, bright field; Scale bar, 20 μm.

We incubated AMPR-22 with mice RBCs, CRPA, or CRPA and mice RBCs for 10 min. When TAMRA-labeled AMPR-22 was incubated at concentrations of 5, 10, or 25 µg/mL in CRPA (Figure 6A) or RBCs (Figure 6B), a 128-fold higher mean value of fluorescence PE-A was shown in CRPA compared to the RBC group at 25 µg/mL. Similarly, the CRPA/RBCs in a 1:1 ratio showed that AMPR-22 bound to bacteria at levels 44- or 102-fold higher than RBCs at concentrations of either 5 or 25 µg/mL (Figure 6C), respectively. For confocal microscopy, 5 µg/mL TAMRA-labeled AMPR-22 was incubated with CRPA, RBC, or CRPA/RBC for 10 min. In the RBC group, AMPR-22 predominantly remained in the solution, whereas AMPR-22 bound specifically to CRPA. In the CRPA/RBC group, AMPR-22 did not bind to RBCs but bound selectively to CRPA (Figure 6D). These results indicate that AMPR-22 targets bacteria specifically without hemolysis in a bacteremia-mimic condition, and this could explain its low toxicity in vivo.

## 3. Discussion

To develop novel AMPs for the treatment of bacteremia or sepsis, we designed AMPR-11 originating from a putative pore-forming region (K58-R78) of Romo1, and we showed that a single dose of AMPR-11 was effective in a murine model of sepsis triggered by either methicillin-resistant *S. aureus* (MRSA) or clinically isolated carbapenem-resistant strains (*P. aeruginosa, K. pneumoniae*, and *A. Baumannii*) [18]. However, the minimum bactericidal concentration of AMPR-11 (100 µg/mL) was not ideal for development of a drug. Therefore, four polar residues of AMPR-11 were replaced with four lysines to construct a more positive net charge, conferring a higher bacterial membrane affinity. The optimized derivative AMPR-22 showed significantly higher antibacterial activity (1–4 µg/mL) than AMPR-11 while maintaining a broad antimicrobial spectrum and a decreased hemolytic toxicity, which is one of the critical side effects in the development of AMPs administered intravenously. 

AMPR-22 has many advantages as an anti-bacteremia/sepsis pharmaceutical. If the patients are hospitalized because of a symptom of sepsis, empiric antibiotics should be intravenously administered before bacterial identification. This is because the survival rate falls to 0% within 36 h if antibiotics are not administered timely (World Federation of Pediatric Intensive & Critical Care Societies). Therefore, there is an urgent need for potent antibiotics with broad-spectrum antimicrobial activity against both Gram-negative and Gram-positive bacteria including XDR strains. Because (1) AMPR-22 can eradicate either Gram-positive or Gram-negative bacteria regardless of MDR without hemolytic toxicity [20], and (2) a high fitness cost is required for resistance acquisition, resulting in no AMPR-22-resistance found in vitro contrasting to chemical antibiotics [20], AMPR-22 or an optimized peptide from AMPR-22 could be used as a first-line antibiotic treatment until the identification of the infectious bacteria.

Many experimental and computational approaches could be applied to optimize AMPs. Although the cost of solid-phase peptide synthesis has been decreasing, it is still expensive to study all the possible synthetic AMP derivatives at the screening stage. Therefore, computational tools have been developed to screen AMPs for either de novo design or optimization [31,32,33]. One of these strategies is an MD simulation that is widely used as a powerful predictive tool to define the antimicrobial mechanism of AMPs at an atomic level [34,35,36]. This also could be applied to structure-based refinement for constructing an optimization strategy and ultimately could reduce the costs for AMP development. However, MD is often inadequate if no experimental data support the simulation results. Yukun et al. reported the validity of MD simulations for AMPs interacting with bacterial membranes by comparing them with known experimental results, suggesting that a combined experimental/computational approach can increase the accuracy of identification of the physiochemical properties of AMPs compared to using MD alone [37]. In this study, we verified the prediction of bacterial membrane preference by MD simulations with in vitro and ex vivo experiments. First, we used artificial liposomes that mimicked bacterial OM, IM, or eukaryotic PM to confirm bacterial membrane affinity and AMPR-11/22-induced membrane permeabilization. AMPR-22 showed higher binding affinity to the bacterial OM/IM than AMPR-11 by interacting with lipid A or cardiolipin preferentially. Second, we confirmed that AMPR-22 can interact specifically with clinically isolated CRPA in the presence of mouse RBCs, whose condition could partially mimic bacteremia. The difference in binding affinity between AMPR-11 and AMPR-22 could explain why a single dose of AMPR-22 resulted in a 100% survival rate, whereas that of AMPR-11 was about 60% in a murine model of sepsis [18,20]. Although MD simulations in this study do not represent Gram-positive bacteria that are encapsulated in polysaccharides, AMPR-11 and AMPR-22 were experimentally shown to pass through the bacterial capsule and/or peptidoglycan wall of MRSA, resulting in bacterial inner membrane permeabilization. This mechanism needs to be studied further.

A limitation in MD simulations could be caused by simplified model membranes such as POPC (eukaryotic plasma membrane), POPG (bacterial membrane), or a mixture of POPC and POPG. For example, CM15 [38], LL-37 [24], and branched AMP B2088 [39] used POPC, POPG, POPC + POPG, or POPE + POPG membranes to mimic target membranes, and all AMPs preferred to interact with bacterial membrane-mimicking model membranes. The construction of these lipid compositions is based on the surface charge difference between the eukaryotic plasma membrane and bacterial membrane. Gram-negative bacteria can cause bad outcomes due to the lipopolysaccharides (LPS) of the OM, which act as an endotoxin [40]. Therefore, the bacterial model membranes representing both bacterial OM and IM need to be considered to evaluate AMP in MD simulations. This also applies to the eukaryotic PM where cholesterol, saturated lipids, and sphingolipids decrease the fluidity of the plasma membrane [41,42]. In this study, we constructed each model membrane with a complexed composition to mimic biological membranes: POPC + POPE + SM + cholesterol for eukaryotic PM, lipid A + PPPE + PVPG for bacterial OM, and PPPE + PVPG + PVCL2 for bacterial IM. We identified how the initial contacts occur between AMP and each model membrane and how the 13PHE was deeply inserted into the hydrophobic region of the membrane in the model membranes. Unfortunately, we were not able to detect penetration of AMPR-22 or pore formation in either the all-atom model for 1 µs (CHARMM36m) or the CG model for 1 ms (martini22p). As AMPR-22-induced membrane permeabilization did not occur immediately after adding AMPs (Figure 5), the penetration/pore-forming events of AMPR-22 might occur at a later stage (e.g., multi-ms to multi-s). Although the lipid compositions we used do not represent a realistic membrane as there are many factors involved, a more realistic biological membrane would increase the understanding of the preference of AMPs to disrupt bacterial membranes over plasma membranes. 

In the present study, we showed how AMPR-11/22 prefer bacterial membrane over eukaryotic plasma membrane at an atomic level and verified their affinity with in vitro and ex vivo experiments. We propose that MD simulation-guided AMP optimization could be a cost-effective strategy, and a further optimized AMP from AMPR-22 could be a potential candidate for treatment of bacterial sepsis caused by XDR.

## 4. Materials and Methods

### 4.1. Molecular Modeling and Molecular Dynamic Simulation

The input structure of AMPR-11 or AMPR-22 was created with Modeller in Chimera. MD simulation of AMPR-11 or AMPR-22 with 60 molecules of dodecylphosphorylcholine (DPC) micelles was generated using CHARMM-GUI with a CHARMM36 force field [43,44]. The AMPR-11 or AMPR-22 structure was energy-minimized, equilibrated, and simulated for 2 µs. The AMPR-11 or AMPR-22 structure in 0.5–2 µs (15,000 frames, 0.1 ns for each frame) was used for clustering with Bitclust [45]. The representative structure of AMPR-11 or AMPR-22 was used in the following MD simulations. AMPR-11 or AMPR-22 was placed onto each model membrane, and the lipid compositions of the membranes used in this study are listed in Table 1. All MD simulations were performed with NAMD 3.0 [46] except CG MD simulation (martini22p force field), which was simulated with GROMACS [47]. PPPE:PVPG:Lipid A = (0,71):(0,25):(32,0), PPPE:PVPG:PVCL2 = 47:12:3, or POPC:POPE:SM:CHOL = 22:11:11:18 was used to mimic Gram-negative bacterial OM, IM, or eukaryotic PM, respectively. All calculations were performed with 150 mM NaCl at 310 K under constant particle number, pressure, and temperature (NPT) conditions with a water (TIP3P) thickness of 20 Å. Negatively charged bacterial membranes were neutralized with Na^+^ ions. Unless otherwise indicated, the default options were used. The multiphase equilibration is composed of six steps including energy minimization in Membrane Builder of CHARMM-GUI were applied to relax the initial system by gradually reducing force constants: cycle 1, 0.5 fs/step for 0.125 ns; cycle 2–3, 1.0 fs/step for 0.125 ns; cycle 4–6, 2 fs/step for 0.5 ns. A 12 Å cutoff for intermolecular interactions was applied both in equilibration and production simulations. Membplugin 1.1 in VMD (visual molecular dynamics) was utilized to calculate the average area of total lipids and membrane thickness [48,49]. A 2 fs/step was used for production. Hydrogen bonds formed between AMPR-11/22 and each model membrane and the distance of AMPR-22 from the bacterial IM were analyzed with UCSF Chimera software [50,51,52]. The alpha-helical proportion was determined via BeStSel server [53].

### 4.2. Chemicals and Peptides

The 16:0–18:1 PE (POPE), 16:0–18:1 PG (POPG), Kdo2-lipid A, 16:0 cardiolipin, and egg sphingomyelin were purchased from Avanti Polar Lipids (Alabaster, AL, USA). L-α-Phosphocholine, L-α-phosphethanolamine, and cholesterol were purchased from Sigma-Aldrich (St. Louis, MO, USA). AMPR-22 with or without TAMRA, TAMRA-labeled AMPR-11, and melittin were chemically synthesized using solid-phase synthesis at GL Biochem (Shanghai, China). Peptides were purified to >75% by high-performance liquid chromatography and verified by mass spectroscopy. All peptides were dissolved in 50% HFIP.

### 4.3. Bacterial Strains

CRPA was clinically isolated in Korea University Hospital (Institutional Review Board, no. 2015AN0129). CRPA was confirmed to be resistant to piperacillin, piperacillin-taxzobactam, ceftazidime, imipenem, meropenem, gentamicin, amikacin, and ciprofloxacin.

### 4.4. Liposome Adhesion Assay

Either 20 µL of 5 mM bacterial OM-mimic lipid mixture (POPE:POPG:Lipid A = 71:25:32, molar ratio), bacterial IM-mimic lipid mixture (POPE:POPG:Cardiolipin = 47:12:3, molar ratio), or eukaryotic PM-mimic lipid mixture (L-α-phosphocholine: L-α-phosphethanolamine:SM:cholesterol = 22:11:11:18, molar ratio) containing 0.025 mM NBD-PE [1,2-dioleoyl-sn-glycero-3-phosphoethanolamine-N-(7-nitro-2-1,3-benzoxadiazol-4-yl)] was dried in a glass vial under a nitrogen stream and rehydrated with 150 mM NaCl and 10 mM HEPES/NaOH (pH 7.4) (rehydration buffer). Multilamellar vesicle (MLV) suspensions were pelleted at 5000 rpm and washed twice with rehydration buffer. MLVs were incubated with 5 µg/mL TAMRA-labeled AMPR-22 for 10 min and observed with confocal laser scanning microscopy (LSM 800, ZEISS).

### 4.5. Liposome Permeabilization Assay

A 5 mM lipid mixture of bacterial OM, IM, or eukaryotic PM was dried and rehydrated with a buffer containing 50 mM CF, 100 mM sucrose, and 5 mM HEPES/NaOH (pH 7.4). MLV suspensions were extruded through a 0.1-µm polycarbonate membrane using a Mini Extruder (Avanti Polar Lipids), and the CF-encapsulated LUVs were purified using a PD-10 desalting column (GE Healthcare) with an external buffer (100 mM NaCl, 100 mM sucrose, 10 mM HEPES/NaOH, pH 7.4). 0.1% HFIP or 1% Triton X-100 was used as a negative or positive control, respectively. CF leakage was measured using a SpectraMax i3x microplate reader (Molecular Devices), and the liposome permeabilization was calculated using the formula: CF leakage (%) = 100 × (F − NC)/(Fmax − NC), where F is the measured fluorescence intensity, NC is the fluorescence intensity of LUV treated with 0.1% HFIP, and Fmax is the fluorescence intensity of LUVs treated with 1% Triton X-100.

### 4.6. The Interaction of AMPR-22 with Bacteria or RBCs

Blood (200 µL) from a male Balb/c mouse was collected via retro-orbital bleeding (Institutional Review Board, no. KOREA-2021-0210) and added to an ethylene-diamine-tetraacetic acid (EDTA)-treated tube. Mouse RBCs were collected by centrifugation at 500× *g* for 10 min and washed three times with phosphate-buffered saline (PBS). The RBCs were kept at room temperature and used within 4 h of isolation. CRPA was cultured with Luria-Bertani medium overnight and re-cultured until O.D. reached 1.0. The interaction of TAMRA-labeled AMPR-22 with CRPA or RBCs was examined using FACS Canto II (BD Biosciences, CA, USA) or confocal laser scanning microscopy (LSM 800, ZEISS, Germany). TAMRA-labeled AMPR-22 was incubated with CRPA (5 × 10^7^ cfu/mL), RBCs (5 × 10^7^ cells/mL), or CRPA (5 × 10^7^ cfu/mL) + RBCs (5 × 10^7^ cells/mL) at room temperature for 10 min before analysis. The data were analyzed with FlowJo software (Tree Star, Inc., Ashland, OR, USA) or ZEN 2 software (Zeiss GMBH, Jena, Germany).

## Figures and Tables

**Table 1:** Summary of molecular dynamics simulation systems.

Force Field	Membranes	Lipid Composition	PLR	Time	Iterations
CHARMM36m	Bacterial OM	Outer leaflet: Lipid A = 32	1/128	10 ns	100
		Inner leaflet: PPPE:PVPG = 71:25			
	Bacterial IM	PPPE:PVPG:PVCL2 = 47:12:3	1/124	10 ns	100
	Eukaryotic PM	POPC:POPE:SM:CHOL = 22:11:11:18	1/124	10 ns	100
	Bacterial IM	PPPE:PVPG:PVCL2 = 47:12:3	1/124	1 µs	3
Martini22p	Bacterial IM	POPE:POPG:CDL2 = 705:180:45	9/1860	1 ms	3

OM, outer membrane; IM, inner membrane; PM, plasma membrane; PLR, peptide lipid ratio; PPPE, 1-palmitoyl-2-palmitoleoyl-phosphatidylethanolamine; PVPG, 1-palmitoyl-2-vacenoyl-phosphatidylglycerol; PVCL2, 1,10-palmitoyl-2,20-vacenoyl cardiolipin; POPC, 1-palmitoyl-2-oleoyl-sn-glycero-3-phosphocholine; POPE, 1-palmitoyl-2-oleoyl-sn-glycero-3-phosphoethanolamine, SM, sphingomyelin; CHOL, cholesterol; POPG (1-palmitoyl-2-oleoyl-sn-glycero-3-phosphoglycerol); CDL2, cardiolipin.

**Table 2:** Lipid preference of AMPR-22.

Membrane Models	Lipid in Outer Leaflet	Ratio	Total Hydrogen Bonds with AMPR-22	Lipid Preference (%)
Bacterial OM	Lipid A	32	26,716	100
Bacterial IM	PPPE	47	15,884	16.32
	PVPG	12	7193	28.94
	PVCL2	3	3401	54.74
Eukaryotic PM	POPC	22	1458	41.20
	POPE	11	753	42.56
	SM	11	280	15.83
	CHOL	18	12	0.41

Lipid preference (%) = hydrogen bonds formed between AMPR-22 and indicated lipids in outer leaflet/ratio ÷ sum of hydrogen bonds/ratio of each lipid in outer leaflet constituting membrane × 100. PPPE, 1-palmitoyl-2-palmitoleoyl-phosphatidylethanolamine; PVPG, 1-palmitoyl-2-vacenoyl-phosphatidylglycerol; PVCL2, 1,10-palmitoyl-2,20-vacenoyl cardiolipin; POPC, 1-palmitoyl-2-oleoyl-sn-glycero-3-phosphocholine; POPE, 1-palmitoyl-2-oleoyl-sn-glycero-3-phosphoethanolamine, SM, sphingomyelin; CHOL, cholesterol.

## Data Availability

The data that support the findings of this study are available from the corresponding author upon reasonable request.

## Supplementary Materials

The following supporting information can be downloaded at: https://www.mdpi.com/article/10.3390/ijms23137404/s1.
