# Supplementary material for: Identification of Bacterial Membrane Selectivity of Romo1-Derived Antimicrobial Peptide AMPR-22 via Molecular Dynamics
Source: Int J Mol Sci. 2022 Jul 3;23(13):7404. doi: 10.3390/ijms23137404 (PMC9266825; doi:10.3390/ijms23137404)
Supplement: Supplementary file 1 [file ijms-23-07404-s001.zip › ijms-1781607-supplementary.pdf]

International Journal of
Molecular Sciences

MDPI

Supplementary Materials

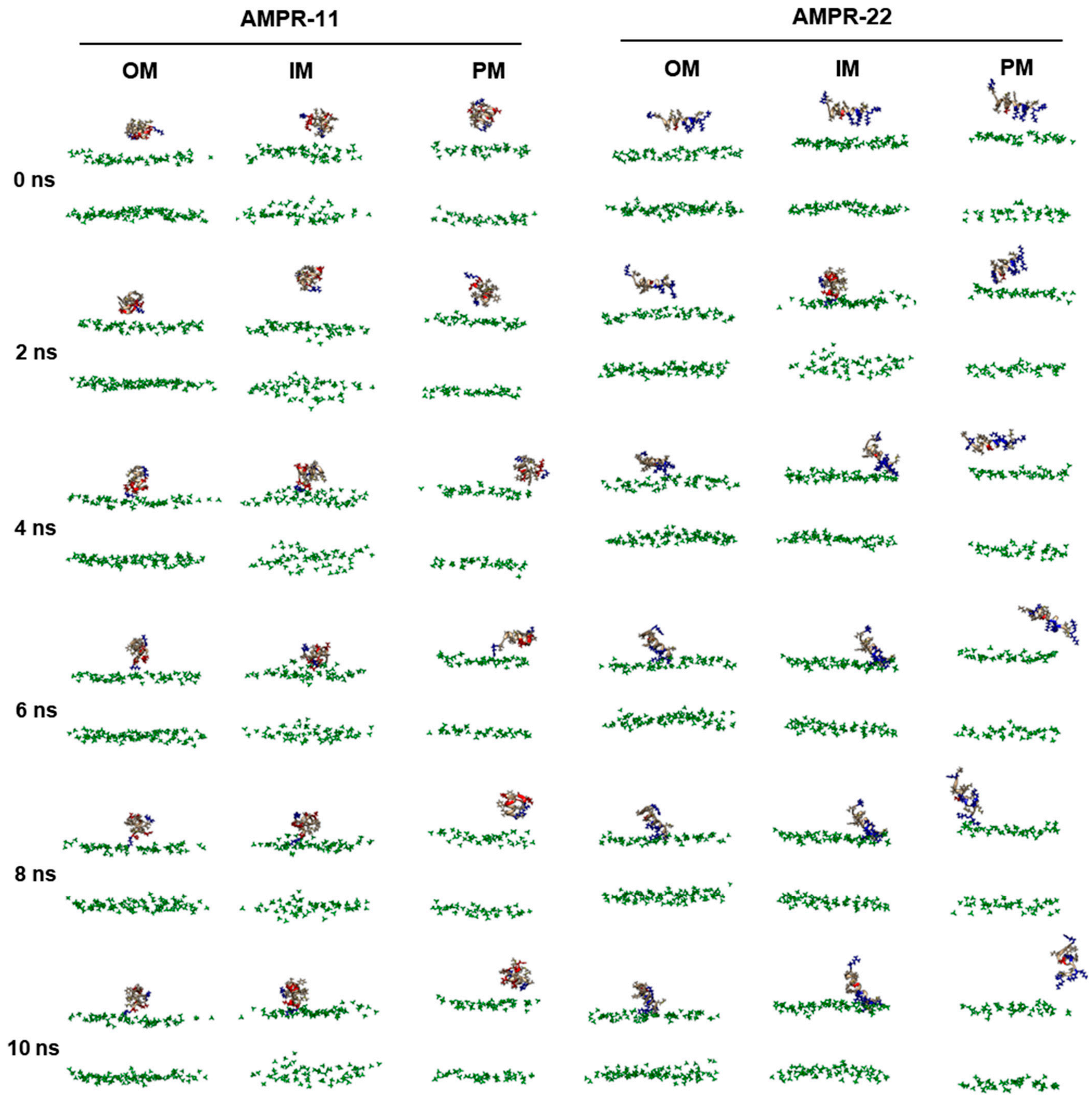

Figure S1. Representative structures of AMPR-11/22 interaction with Gram-negative bacterial outer/inner membrane and eukaryotic plasma membrane model. Phosphate group of the lipid is colored green. Hydrophobic (nonpolar), hydrophilic (polar), and cationic residues of AMPR-11 are shown in copper, red, and blue, respectively.

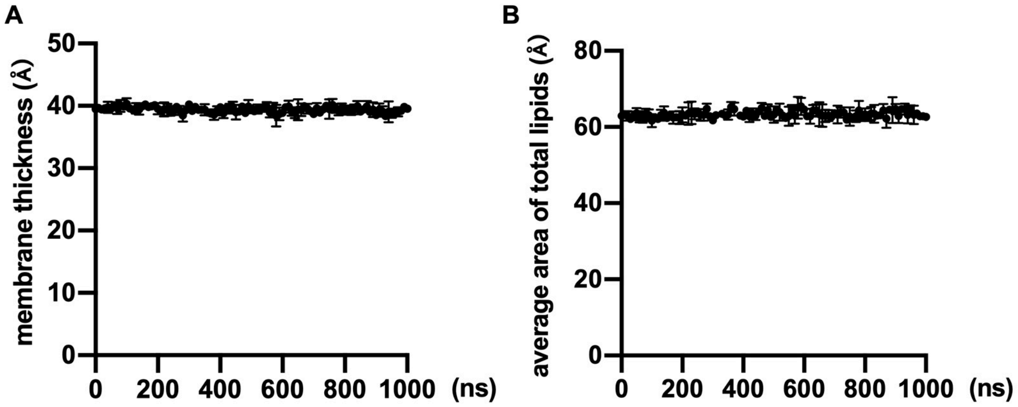

Figure S2. Average area of total lipids (A) and membrane thickness (B) of bacterial inner membrane during 1 μs simulations were calculated with 3-carbon backbone (C1, C2, and C3) of glycerol in each lipid.

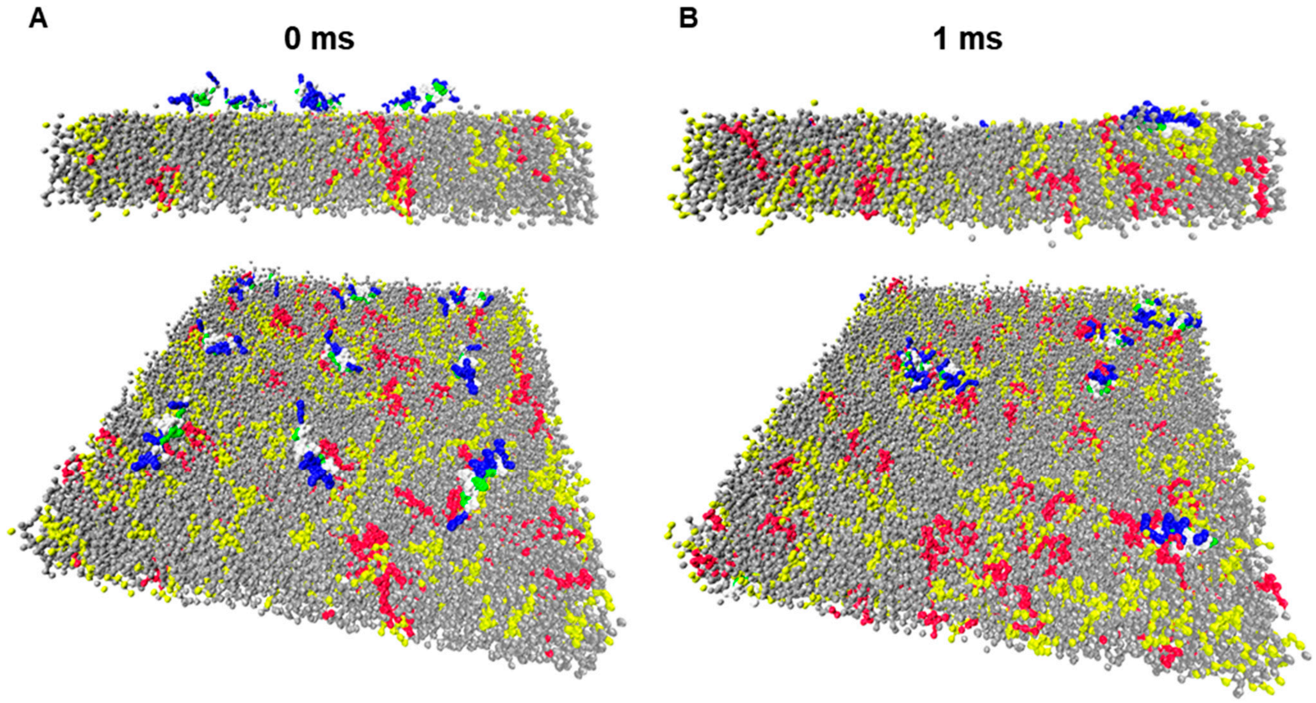

Figure S3. Coarse-grained MD simulation of AMPR-22. Nine AMPR-22 molecules were placed onto the bacterial inner membrane and simulated for 1 ms under a martini22p force field. POPG, POPE and CDL2 of the lipid are colored yellow, gray, and red, respectively. Hydrophobic (nonpolar), hydrophilic (polar), and cationic residues of AMPR-22 are shown in white, green, and blue, respectively.
